# Pulmonary Large-Cell Neuroendocrine Carcinoma, a Multifaceted Disease—Case Report and Literature Review

**DOI:** 10.3390/diagnostics15091056

**Published:** 2025-04-22

**Authors:** Ancuța-Alina Constantin, Antonio Andrei Cotea, Florin-Dumitru Mihălțan

**Affiliations:** 1 Institute of Pneumology “Marius Nasta”, 050159 Bucharest, Romania; andrei.cotea@stud.umfcd.ro (A.A.C.);; 2 Department of Cardio-Thoracic Pathology, “Carol Davila” University of Medicine and Pharmacy, 050474 Bucharest, Romania

**Keywords:** large-cell neuroendocrine carcinoma, asthma, wheezing, differential diagnosis, bronchoscopy

## Abstract

**Background and Clinical Significance:** This article explores the complexity of large-cell neuroendocrine carcinoma (LCNEC) by presenting a clinical case involving a 17-year-old admitted for persistent wheezing, with no history of respiratory toxin exposure, a background of atopy, and a suspected diagnosis of bronchial asthma. Given the patient’s age and the nature of the symptoms, the condition was initially diagnosed as asthma, leading to the initiation of maximum inhalation therapy. **Case Presentation:** Despite proper adherence and correct administration, symptoms persisted, necessitating the use of oral corticosteroids. Imaging revealed an extensive inhomogeneous mass in the cervical esophagus and trachea, along with a similar tumor in the right hilum, prompting bronchoscopy. The diagnosis of LCNEC was confirmed through imaging, histopathological findings, and a detailed immunohistochemical profile. Initially misdiagnosed as adenoid cystic carcinoma, this case highlights the diagnostic challenges and the importance of rigorous evaluation. **Conclusions:** It emphasizes that recurrent wheezing in adolescents is not always indicative of asthma and requires careful differential diagnosis to uncover less common causes.

## 1. Introduction

Large-cell neuroendocrine carcinomas (LCNECs) are a rare subtype of lung cancer, accounting for approximately 15% of neuroendocrine tumors and about 3% of all lung cancers [1]. Classified as poorly differentiated, high-grade neuroendocrine tumors, LCNECs lie between atypical carcinoid tumors and small-cell lung carcinoma (SCLC) in terms of morphology and biology [2]. Patients often present with minimal symptoms, such as chest pain, dyspnea, flu-like symptoms, or night sweats [3]. Treatment strategies for LCNEC vary depending on the disease stage. Advanced cases are typically managed with systemic chemotherapy (ChT) and radiotherapy, whereas early-stage disease is treated with surgical resection followed by adjuvant chemotherapy to minimize the risk of recurrence [4]. Histopathologically, LCNEC is characterized by a neuroendocrine growth pattern, including organoid clusters, nests, trabeculae, palisading cells, or rosette formations, along with immunohistochemical evidence of neuroendocrine differentiation [5]. This aggressive tumor is associated with poor prognosis due to its rapid progression and the challenges associated with treatment [6]. The biological complexity of LCNEC reflects similarities to both SCLC and non-small-cell lung cancer (NSCLC) [7].

The 2015 WHO classification categorizes lung neuroendocrine tumors into four primary types: typical carcinoids, atypical carcinoids (both classified as low-grade tumors), SCLC, and LCNEC. According to the WHO, LCNEC is a morphologically distinct subtype of NSCLC that exhibits histopathological characteristics of neuroendocrine tumors and expresses neuroendocrine markers on immunohistochemical analysis [8]. Similar to SCLC, LCNEC primarily affects older male patients (median age of 65 years) with a history of heavy smoking. However, unlike SCLC, which is predominantly located centrally in the lungs, LCNEC tumors are more often found in the lung periphery. Despite surgical resection in early-stage cases, recurrence rates remain high, generally exceeding 50%, with brain metastases being a frequent complication [9].

Diagnosing LCNEC requires a thorough histopathological evaluation of neuroendocrine features under light microscopy, followed by confirmation via immunohistochemical staining for neuroendocrine markers [3]. Given its aggressive nature and high recurrence rates, clinicians must maintain a high degree of vigilance in diagnosing and managing LCNEC. Additionally, recurrent wheezing—a symptom often linked to bronchiolitis or asthma—can mimic multiple conditions. In atypical presentations, clinicians must carefully assess underlying etiologies to ensure accurate diagnosis and effective management.

## 2. Case Report

**Figure 1:**
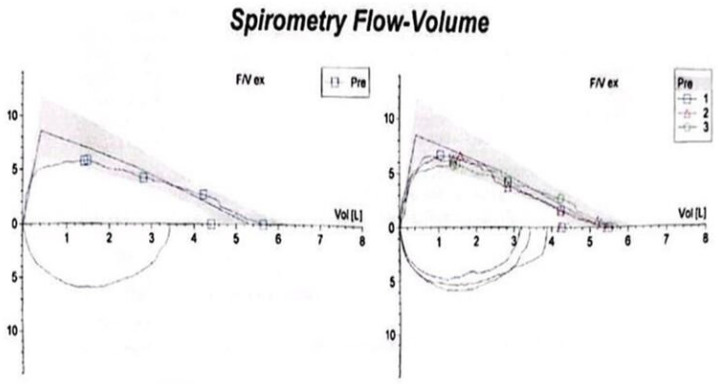
Spirometry showing a fixed upper airway obstruction pattern, with flattening of both the inspiratory and expiratory limbs of the flow–volume loop, consistent with significant tracheal narrowing.

**Figure 2:**
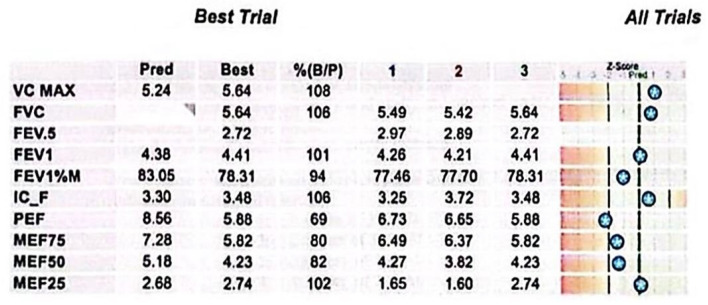
Spirometry results. Key parameters include elevated VC MAX and FVC values, normal FEV1, and a slightly reduced FEV1/FVC ratio. PEF and mid-expiratory flows demonstrate variability, while MEF25 exceeds predicted levels, indicating preserved distal airway function. The Z-score bars display each test result’s deviation from predicted normal values, based on the patient’s demographic profile. Stars mark the measured values along a scale ranging from −5 to +3. Clinically, results falling within ±1.64 Z-score are considered within the normal range; values beyond this suggest potential respiratory impairment.

This case involves a 17-year-old high-performance athlete, a non-smoking male, who was initially misdiagnosed with asthma due to persistent wheezing—an atypical symptom for his age. Despite strict adherence to maximum inhalation therapy and the addition of oral corticosteroids, his symptoms persisted. Over time, his condition worsened, progressing from wheezing to exertional shortness of breath and dyspnea with minimal effort. Additionally, he reported stridor, bilateral supraclavicular retraction, and dysphagia. His medical history included multiple documented allergies and allergic rhinitis, with no known exposure to occupational or domestic respiratory irritants. A previous spirometry demonstrated a fixed upper airway obstruction pattern, characterized by flattening of both the inspiratory and expiratory limbs of the flow–volume loop, consistent with significant tracheal narrowing (Figure 1). Follow-up spirometry (Figure 2) showed normal-to-above-average lung volumes, with a maximum vital capacity (VC MAX) of 5.64 L (108%) and a forced vital capacity (FVC) of 5.64 L (106%). The close agreement between VC MAX and FVC suggests no significant dynamic airway collapse or air trapping—a finding typical of fixed upper airway obstruction, such as tracheal stenosis, where flow limitation occurs during both inspiration and expiration but lung volumes remain preserved. Peak expiratory flow (PEF) and mid-expiratory flows showed some variability, with MEF25 slightly elevated (102%), likely reflecting normal physiological variation rather than small airway pathology. Overall, these findings indicate preserved lower airway function, with the flow limitation primarily attributable to the fixed upper airway obstruction. It is important to mention that the spirometry was performed in a medical facility located in the patient’s county of residence. After admission to our unit, considering the patient’s clinical condition marked by severe dyspnea, it was no longer feasible to perform this investigation.

**Figure 3:**
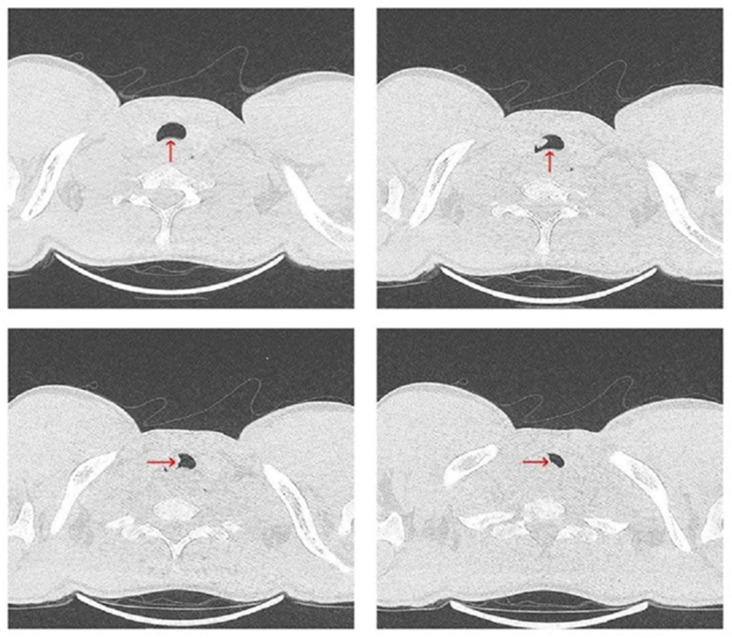
Chest CT scan from 19 July 2024—axial plane, showing a heterogeneous mass (red arrow), stenosing more than 50% of the trachea.

**Figure 4:**
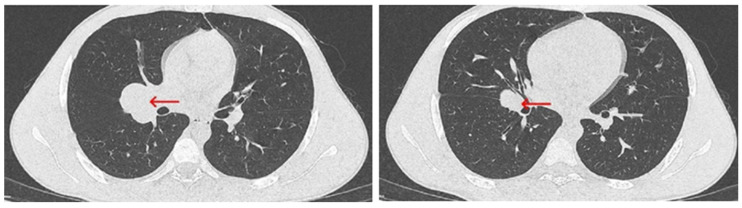
Chest CT scan from 19 July 2024—axial plane, identifying a mass measuring 43 × 30 mm in the right pulmonary hilum (red arrow).

Upon admission, the patient was referred for a chest CT scan, which revealed a heterogeneous mass measuring 26 × 50 × 49 mm. The mass extended from the hypopharynx, infiltrating the cervical esophagus and trachea, resulting in over 50% tracheal stenosis (Figure 3). Additionally, a separate mass measuring 43 × 30 mm was identified in the right pulmonary hilum (Figure 4). These findings were associated with inspiratory stridor, prompting his urgent transfer to intensive care for continuous monitoring. Initial laboratory investigations demonstrated mild hypochloremia, hyperglycemia, neutrophilic leukocytosis, low fibrinogen levels, and a slight elevation in hemoglobin concentration (Table 1).

**Figure 5:**
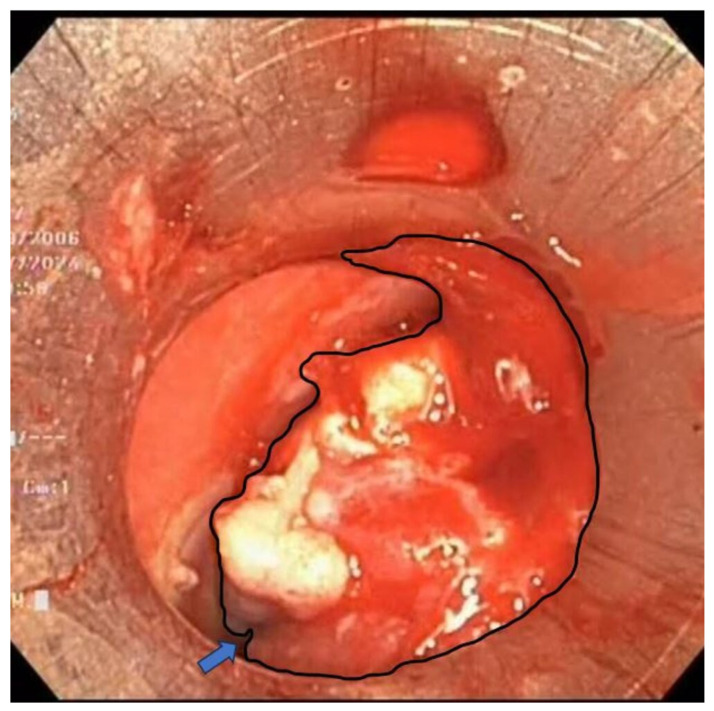
An iceberg-like infiltrative mass (outlined by the black contour) is noted, causing near-complete obstruction of the tracheal lumen, with only a very narrow residual passage (blue arrow) permitting limited airflow.

Rigid and flexible bronchoscopy, performed under general anesthesia, revealed extrinsic compression and an iceberg-like infiltrative mass causing near-complete tracheal obstruction, with the remaining luminal narrowing reduced to just 5 mm (Figure 5).

**Figure 6:**
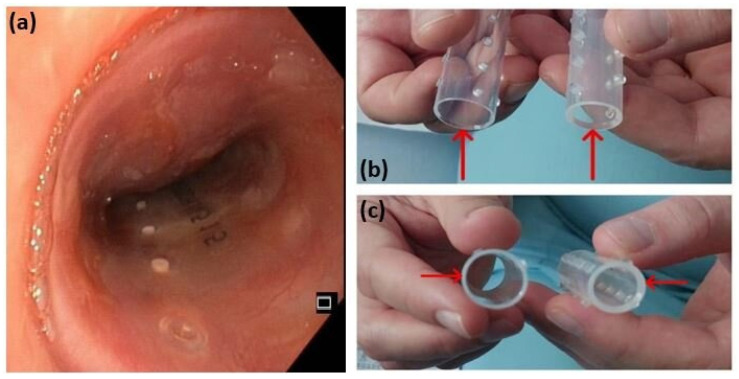
A Dumon stent (**a**); comparison between the two Dumon stents of different wall thickness (**b**,**c**). The initial stent, on the left, with a diameter of 18 mm and a wall thickness of 1 mm, was replaced with the stent on the right, with a diameter of 16 mm and a wall thickness of 1.5 mm, to address persistent tracheal instability.

A partial resection of the mass was performed using diathermy and mechanical excision. Histopathological analysis of the resected tissue initially suggested a poorly differentiated squamous cell carcinoma. To maintain airway patency, a Dumon stent was inserted (Figure 6a). However, due to persistent tracheal instability, the initial stent was later replaced with one of greater wall thickness (Figure 6b).

During bronchoscopy, a *Staphylococcus aureus* infection was identified, with an antibiogram guiding the treatment strategy. The strain was determined to be methicillin-sensitive, making it susceptible to beta-lactam antibiotics, which are commonly used for staphylococcal infections. Following these interventions, the patient’s respiratory status improved significantly, and he was transferred to the thoracic surgery ward in stable condition for continued care.

**Figure 7:**
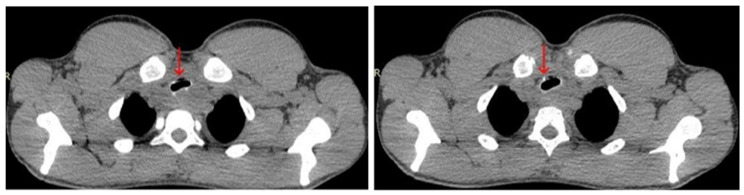
Chest CT scan from 24 July 2024—axial plane, with contrast, showing a residual mass (red arrow) compressing the trachea and esophagus, appearing hypo-iodophilic and heterogeneous, likely adenopathic or tumor-related.

**Figure 8:**
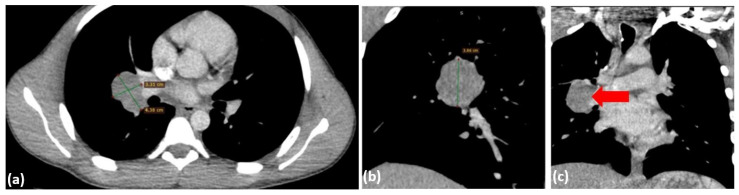
Chest CT scan with contrast from 24 July 2024—axial plane (**a**) and coronal plane (**b**,**c**), identifying a polynodular, well-demarcated mass in the right pulmonary hilum, measuring 4.38 × 3.31 cm (red arrow).

The patient’s post-procedural course was favorable, with stabilization of respiratory function and no evidence of hypoxemia, either at rest or with moderate effort. A follow-up CT scan was performed, partially correlating with the initial imaging findings. The scan revealed a residual tumor mass measuring 5.7 × 3.2 × 5.1 cm, located posterior to the tracheal lumen, near the site of the prior tumor resection and stenting. This mass exerted significant compressive effects on the trachea, reducing the minimal luminal width to 22 × 4 mm, and also affected the esophagus (Figure 7). In the right pulmonary hilum, a polynodular, well-defined mass measuring 4.38 × 3.31 cm was identified, causing mild compression of the regional bronchi (Figure 8), suggesting possible adenopathy. Pulmonary vessels are patent, with isolated perihilar ground-glass opacities and resolved pneumomediastinum.

The patient was discharged with recommendations for oncological evaluation, radiotherapy planning, and periodic bronchoscopic re-assessment to monitor the stent and ensure airway patency.

**Figure 9:**
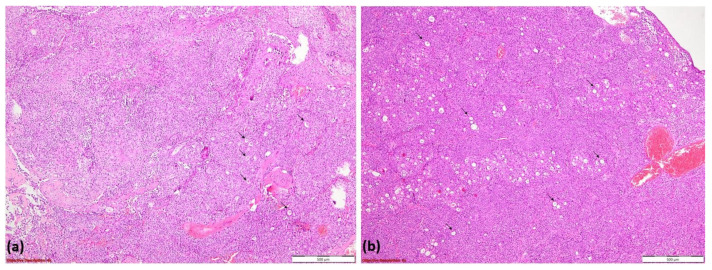
Hypercellular tumoral proliferation with a solid pattern of growth and zonal luminal development (→); HE, 40× (**a**); tumoral nests composed of large neoplastic cells with eosinophilic or clear cytoplasm and vesicular nuclei. Tumoral nests are divided by delicate fibro-vascular septa (→); HE, 100× (**b**).

Histopathological examination revealed basaloid tumor cells with high mitotic activity and a tubular pattern at the periphery, consistent with an initial diagnosis of adenoid cystic carcinoma (ACC). The tumor exhibited hypercellular proliferation with a solid growth pattern and zonal luminal development (Figure 9a). It consisted of large neoplastic cells with eosinophilic or clear cytoplasm and vesicular nuclei, organized into tumoral nests separated by delicate fibrovascular septa (Figure 9b).

Immunohistochemical (IHC) profiling confirmed high-grade ACC, demonstrating preserved mismatch repair (MMR) proteins and the absence of HER2 and p53 overexpression. Molecular testing ruled out mutations in EGFR, ALK, ROS1, and NTRK genes.

A multidisciplinary team, including pulmonologists, thoracic surgeons, oncologists, radiologists, and pathologists, reviewed the case. The tumor was deemed inoperable due to extensive mediastinal involvement and significant tracheal compression. A PET-CT scan revealed a metabolically active mass in the cervico-mediastinal region with a standardized uptake value (SUV) of 5.63. The tumor was found to be infiltrating the esophagus and compressing the trachea.

Subsequent pathological examination of the biopsy identified compact, dense tumor cell nests with mitotic rates of up to 10 mitoses per 10 high-power fields (HPFs). Immunohistochemical profiling (Table 2) further supported the diagnosis, with several markers, including Pan-cytokeratin, CK7, Synaptophysin, CD117, and PAX8, being positive, while others, such as CK5/6, p63, and Chromogranin A, were negative.

The clinicopathological interpretation ruled out germ cell tumors, as evidenced by the absence of CD30, PLAP, and Beta-HCG expression. Additionally, the lack of p63, S-100, and CK5/6 expression excluded the possibility of ACC. Furthermore, negative results for thyroglobulin, TTF1, and calcitonin ruled out a thyroid-origin tumor.

Based on these findings, the diagnosis was confirmed as large-cell neuroendocrine carcinoma (LCNEC), grade 3 (G3), effectively ruling out the earlier suspicion of ACC, which had been considered based on imaging features.

**Figure 10:**
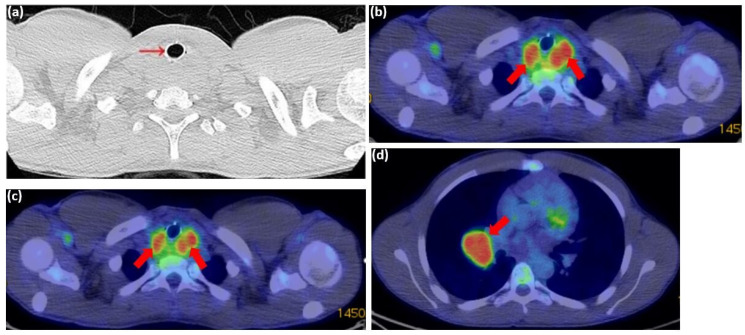
PET-CT scan from 31 July 2024: (**a**) axial plane showing post-stenting recalibration of the tracheal lumen (red arrow); (**b**–**d**) axial plane showing a hypermetabolic tumor mass in the cervico-mediastinal posterior region, measuring approximately 55 × 65 mm axially and 45 mm cranio-caudally (red arrows). The lesion is inseparable from the posterior thyroid lobes; (**d**) the axial plane identifies a metabolically active, well-defined mass in the right pulmonary hilum, consistent with adenopathy (red arrows).

One month later, a PET-CT scan revealed a highly hypermetabolic tumor mass in the posterior cervico-mediastinal region, measuring approximately 55 × 65 mm axially and 45 mm craniocaudally (Figure 10a–c), with a standardized uptake value (SUV) of 5.63. The lesion was inseparable from the posterior thyroid lobes and had encased the cervical esophagus and trachea, which contained a stent. Additionally, a metabolically active, well-defined mass was identified in the right pulmonary hilum, consistent with adenopathy (Figure 10d). No pleural or pericardial effusions or other significant lesions were detected on this scan.

Given the aggressive nature of the tumor and its mediastinal lymph node involvement, the patient was started on induction chemotherapy with cisplatin and etoposide. Supportive medications included antiemetics and hydration therapy. The initial treatment was well tolerated, with plans to continue chemotherapy in 21-day cycles. To reduce the risk of neutropenia, prophylactic growth factor support was provided using pegfilgrastim. Despite being an athlete, the patient was advised to limit physical activity due to the cardiotoxic and nephrotoxic effects of chemotherapy. His overall condition improved post-intervention, with resolution of acute dyspnea. As of the latest follow-up, the patient remains clinically stable and continues to receive ongoing oncological treatment. He is currently under regular monitoring by the oncology team.

Several challenges emerged in managing this case. LCNEC is a high-grade malignancy with rapid progression, poor response to treatment, and limited therapeutic options beyond conventional chemotherapy. The tumor’s biology presents a significant challenge, as its aggressive nature limits long-term treatment success. Another key factor was airway management. Tracheal involvement necessitated repeated stent revisions to maintain airway patency, highlighting the complex interplay between tumor burden and airway integrity. In addition, the presence of mediastinal lymph node involvement and retrotracheal mass compression emphasized the tumor’s metastatic potential and the complications associated with its progression.

The prognosis remains guarded due to the advanced stage of the disease (IIIB, pT2N3M0) and the current lack of effective targeted therapies. Regular follow-ups, imaging, and molecular profiling updates will be critical to adjusting treatment strategies as needed. A multidisciplinary approach ensures that management remains comprehensive and tailored to the patient’s complex needs.

This case highlights the diagnostic challenges posed by non-specific respiratory symptoms in adolescents, particularly when standard treatments for common conditions like asthma fail. It underscores the importance of a thorough differential diagnosis to identify less common underlying causes. Immunohistochemical analysis played a crucial role in confirming the tumor type, guiding therapeutic decisions, and ultimately influencing the patient’s clinical trajectory.

## 3. Literature Review

Bronchopulmonary neuroendocrine tumors (BP-NETs), accounting for approximately 20% of lung cancers, originate from neuroendocrine cells within the bronchopulmonary epithelium. This group includes typical carcinoid (TC), atypical carcinoid (AC), large-cell neuroendocrine carcinoma (LCNEC), and small-cell lung carcinoma (SCLC), each exhibiting distinct biological behavior despite overlapping structural and immunohistochemical characteristics [10]. Prognostically, typical carcinoid tumors demonstrate low metastatic potential and favorable surgical outcomes, whereas small-cell carcinomas tend to metastasize early, leading to a poorer prognosis [11].

A significant breakthrough in lung cancer diagnosis and treatment has been the advent of personalized medicine, which tailors therapeutic strategies based on a tumor’s histological and genetic profile. This has enhanced the role of pathologists in subclassifying non-small-cell lung cancer (NSCLC), where distinctions such as adenocarcinoma and squamous cell carcinoma now influence molecular testing and targeted therapies—an approach that was previously inapplicable to small tissue samples [12].

Pulmonary neuroendocrine tumors were initially classified into three categories—TC, AC, and SCLC—in the 1970s [13]. Most pulmonary carcinoids are centrally located in the lung and possess metastatic potential [14]. Travis et al. later introduced LCNEC as a distinct fourth category following a detailed analysis of 35 cases, thereby refining and expanding the traditional three-tier classification of pulmonary neuroendocrine (NE) tumors [15]. LCNEC and SCLC predominantly affect heavy smokers around 65 years of age and are frequently diagnosed at a metastatic stage. Both malignancies are highly aggressive, with a 5-year survival rate below 15–25% for LCNEC and approximately 5% for extensive-stage SCLC. Treatment strategies remain largely unchanged, with SCLC being managed through platinum–etoposide chemotherapy, while LCNEC is typically treated using NSCLC-based therapeutic approaches [16]. The incidence of LCNEC diagnoses has increased in recent years, with its metastatic pattern and overall survival closely resembling those of SCLC. However, treatment strategies for early-stage cases appear to align more closely with those of squamous cell carcinoma and adenocarcinoma [5].

Most available treatment data for LCNEC are derived from retrospective studies or small case series. Prognosis remains poor, with surgical intervention recommended for early-stage cases, though it is often insufficient. Platinum-based adjuvant chemotherapy may provide some benefit, while the efficacy of neoadjuvant therapy remains uncertain. For advanced-stage disease, SCLC-based chemotherapy remains the standard approach, though with limited therapeutic success [6].

### 3.1. Pathological Diagnosis and Molecular Features

In the 2015 WHO classification of thoracic tumors, LCNEC is categorized alongside other neuroendocrine (NE) lung tumors [9]. The current diagnostic algorithm for pulmonary neuroendocrine neoplasms (NENs) is based on morphological criteria established in the early 1990s for LCNEC, as well as earlier criteria for carcinoid tumors and SCLC [17].

Diagnosing LCNEC is often challenging and typically requires a large, surgically resected lung biopsy. Smaller biopsy samples may be prone to crushing artifacts, which can distort neuroendocrine morphology and cell size, complicating accurate diagnosis [18]. A definitive diagnosis necessitates a thorough evaluation of neuroendocrine features using light microscopy, supported by immunohistochemical (IHC) staining for neuroendocrine markers [3]. The diagnostic criteria include cytological features characteristic of non-small-cell carcinoma, such as prominent nucleoli and large cell size, combined with NE architectural patterns like organoid nesting and peripheral palisading. Essential IHC markers include synaptophysin (SNP), chromogranin (Ch), and CD56, in addition to evidence of high mitotic activity [9]. LCNEC typically expresses multiple NE markers, and any level of NE marker positivity, in combination with clear NE morphology, supports the diagnosis. While electron microscopy can detect NE granules, IHC is now the standard diagnostic approach. A comprehensive pathological review is recommended to avoid misclassification, as LCNEC can resemble poorly differentiated NSCLC, adenocarcinoma, or even SCLC. Small biopsy or cytological samples often lack definitive features and may be classified as non-small-cell lung carcinoma—not otherwise specified (NSCLC-NOS).

The two most frequently used IHC markers for NE differentiation are chromogranin (Ch) and synaptophysin (SNP), as they demonstrate the strongest correlation with ultrastructural NE differentiation. Additionally, neural cell adhesion molecule (N-CAM) is another marker used for NE differentiation, offering good sensitivity and specificity [19].

Differentiating pulmonary neuroendocrine carcinoma (NEC) from other similar tumors is complex, but key criteria aid in classification. Atypical carcinoid tumors exhibit 2–10 mitoses per 2 mm^2^, whereas high-grade NECs surpass 11 mitoses per 2 mm^2^. Necrosis is focal in atypical carcinoid tumors but extensive in LCNEC. Cytological examination distinguishes LCNEC from SCLC, as SCLC cells are smaller, have a higher nuclear-to-cytoplasmic ratio, and exhibit faint or absent nucleoli. However, cell size variation and mitotic count alone have limited diagnostic utility [18].

Imaging characteristics of LCNEC typically show it as a well-circumscribed, lobulated mass without air bronchograms or calcifications. Larger tumors may exhibit heterogeneous enhancement due to necrosis, although this is less pronounced in smaller tumors. Pulmonary carcinoid tumors occur more frequently in the right lung (3:2) and upper lobes [2]. A study by Kyung Won Lee et al. found that LCNEC tumors are more commonly located in the lung periphery, often presenting with well-defined, lobulated margins on CT scans. Additionally, a high mean SUVmax (Standardized Uptake Value maximum) was associated with malignancy, while poor prognosis correlated with female sex, larger tumor size, non-lobulated margins, and higher SUVmax [20].

A distinguishing characteristic of LCNEC, compared to typical and atypical bronchial carcinoids, is its high expression of glucose transporter 1 (GLUT1). This property makes LCNEC well-suited for fluorine-18-fluorodeoxyglucose ([18F]FDG) imaging. As a result, whole-body staging in LCNEC is performed using [18F]FDG PET/CT, with a particular focus on mediastinal N-staging and detecting distant metastases [4].

### 3.2. Prognostic Factors

LCNEC demonstrates poor survival outcomes, similar to SCLC, with high rates of lymph node involvement (60–80%) and distant metastases (40%) at diagnosis. The five-year survival rate ranges from 15% to 57%, decreasing to 30% in cases of combined LCNEC. Even in resectable stage I cases, survival is between 27% and 67%, with frequent recurrences occurring within two years. The disease is predominantly associated with male sex, older age (median: 65 years), and heavy smoking [8].

Patients with LCNEC tend to have poorer overall survival, particularly those with advanced age (>80 years), male gender, larger tumor size (20–29 mm, 40–49 mm, and ≥50 mm), lymph node involvement, metastases (e.g., liver metastases), advanced disease stages (II–IV compared to stage I), and those who do not undergo surgery at the primary tumor site [21,22].

A retrospective analysis of LCNEC patients treated with curative intent across three institutions between May 2005 and January 2017 identified lymphatic invasion (L1) as an independent prognostic factor, reinforcing the benefits of surgery in early-stage LCNEC. Additionally, platinum-based adjuvant chemotherapy may improve long-term outcomes, particularly in stage Ib cases [23].

Regarding long-term survival and recurrence-free survival (RFS), the authors of one study found that these outcomes were primarily influenced by pathologic nodal status and individual neuroendocrine marker profiles, rather than patient age, sex, or the administration of adjuvant therapy [24].

### 3.3. Treatment

#### 3.3.1. Early-Stage Disease

Despite its similarities with SCLC, surgery remains the cornerstone of treatment for localized LCNEC. However, due to the rarity of this NSCLC subtype, the quality of available data is limited, with most recommendations relying on retrospective studies. Nonetheless, surgery continues to be the primary treatment for resectable LCNEC, despite the lack of strong supporting evidence [25]. LCNEC patients accounted for 3.5% of those undergoing surgery for pulmonary neoplasms, a slightly higher percentage than previously reported. Their survival rates were 60.4% at 1 year, 27.5% at 3 years, and 21.2% at 5 years, which are lower than NSCLC survival rates but similar to SCLC outcomes [26]. Post-surgical recurrence is common in LCNEC patients, and those who do not experience recurrence often develop metachronous second primary cancers [27]. Therefore, surgical resection alone is insufficient to significantly improve prognosis in patients with large-cell carcinoma exhibiting neuroendocrine features [28].

Platinum-based adjuvant chemotherapy administered post-surgery may help reduce recurrence rates. Furthermore, multivariate analyses indicate that platinum-based adjuvant chemotherapy significantly impacts outcomes in LCNEC patients [27]. A study by Akira I. et al. found that advanced-stage large-cell carcinoma with neuroendocrine features has a poor prognosis and does not benefit from cisplatin, carboplatin, or cyclophosphamide adjuvant chemotherapy. However, for early-stage LCNEC patients, adjuvant chemotherapy may improve overall survival [28].

Accurate staging is essential before performing thoracic resection with curative intent, particularly in evaluating lymph node involvement and distant metastasis [4]. To achieve a better prognosis, the optimal treatment for stage I, II, and III LCNEC patients is surgery combined with chemotherapy. In contrast, for stage IV patients, chemotherapy alone is preferred over other treatments, such as surgery or radiation therapy [29]. Among LCNEC patients who undergo complete resection, recurrence—particularly in the form of distant metastases—is highly common. As a result, surgery alone is often insufficient, and additional adjuvant therapy is frequently required [30]. In a large-scale analysis by Kujitan et al., which included over 1200 stage I LCNEC patients, the authors found that chemotherapy, primarily administered post-surgery, was associated with improved overall survival. This benefit was observed in both stage IA and IB patients and remained statistically significant even after adjustments, including propensity score matching [31].

#### 3.3.2. Locally-Advanced Disease

For stage IIIA LCNEC, surgery provides a significant survival advantage compared to chemoradiotherapy alone. However, adding a third treatment modality does not further improve outcomes, underscoring the importance of surgery in this stage [32].

Several retrospective studies suggest that LCNEC exhibits a response rate to cisplatin-based chemotherapy similar to that of SCLC, indicating comparable effectiveness of this treatment approach in both cancer types [33]. The authors of one study found that HGNEC-probable LCNEC and SCLC had similar initial response rates and survival outcomes but differed in their response to second-line chemotherapy, suggesting variations in treatment efficacy between the two [34]. A retrospective analysis of 5797 patients reported an increasing use of chemoradiotherapy for LCNEC. Patients receiving this combination treatment showed better overall survival compared to those treated with chemotherapy alone, highlighting the potential benefit of incorporating radiotherapy in LCNEC management [35].

In limited-stage SCLC, combining chemotherapy with thoracic radiotherapy results in complete response rates of 50–85% and a median survival of 12–20 months, with 5-year survival exceeding 20% in complete responders. While this approach reduces thoracic recurrence, brain metastases become the primary site of relapse. In such cases, prophylactic cranial irradiation (PCI) has been shown to improve both overall and disease-free survival in patients who achieve complete remission [36]. Similarly, in locally advanced NSCLC, the combination of chemotherapy, radiation, and surgery can improve survival but also significantly increases the risk of brain metastases (up to 55%), resembling patterns seen in SCLC. Although PCI has been shown to reduce the incidence of brain metastases, its impact on overall survival and quality of life remains under investigation [37].

#### 3.3.3. Advanced Disease

For patients with locally advanced or metastatic LCNEC (stage III or IV), treatment follows standard approaches used for non-SCC (squamous cell carcinoma) and SCC: chemoradiation and chemotherapy for stage III, and chemotherapy with palliative radiation for stage IV [38]. Platinum-doublet chemotherapy regimens, particularly platinum-etoposide combinations, have been shown to significantly improve survival in LCNEC patients. This benefit is observed across different treatment settings, demonstrating favorable outcomes in both the adjuvant setting and metastatic disease [39]. Although LCNEC’s genomic profile and prognosis suggest that it may respond best to SCLC-based treatments, its actual response to SCLC chemotherapy is lower than expected. Phase II trials reported objective response rates of 31–47% for LCNEC, compared to 66% in SCLC trials. Due to this relative resistance to SCLC chemotherapy, some clinicians prefer NSCLC-type regimens [40].

The ASCO guidelines recommend platinum–etoposide chemotherapy (SCLC-PE) or NSCLC-type chemotherapy (for non-squamous NSCLC) for treating LCNEC, with SCLC-PE being the preferred choice. However, recent studies suggest that platinum–gemcitabine or taxane-based chemotherapy (NSCLC-GEM/TAX) may provide better survival outcomes compared to SCLC-PE in LCNEC patients [41]. A study from the Netherlands analyzed 128 LCNEC patients treated with different chemotherapy regimens. Among them, 46% (60 patients) received NSCLC-type chemotherapy (gemcitabine, docetaxel, paclitaxel, or vinorelbine), 16% (20 patients) received NSCLC-pt treatment (pemetrexed only), and 38% (48 patients) received SCLC-type chemotherapy (etoposide). The results indicated that NSCLC-specific regimens, particularly platinum–gemcitabine, were more effective than platinum–pemetrexed and platinum–etoposide, supporting their use as a preferred approach for metastatic LCNEC [40].

A significant proportion of LCNEC cases overexpress the KIT receptor tyrosine kinase (RTK). The KIT signaling pathway is a well-established autocrine growth loop that promotes tumor proliferation and inhibits apoptosis in SCLC. However, the role of RTKs in LCNEC remains poorly understood [42].

Third-generation cytotoxic agents such as paclitaxel, docetaxel, and gemcitabine have been shown to improve survival in advanced lung cancer when combined with platinum-based chemotherapy. Studies suggest that this approach may be as effective for LCNEC as it is for SCLC [43]. Combination chemotherapy using irinotecan (CPT) and cisplatin (CDDP) has demonstrated efficacy in treating both NSCLC and SCLC. While this regimen has also shown activity in LCNEC, the response rate (RR) and overall survival were lower compared to SCLC patients [44].

In conclusion, the optimal first-line treatment for metastatic LCNEC remains uncertain, as most available studies are small, retrospective, and often present conflicting results. Further prospective trials are needed to define the most effective therapeutic approach.

## 4. Discussion

Accurate diagnosis of LCNEC of the lung is crucial, emphasizing the need for heightened clinical vigilance and improved diagnostic protocols. Due to its overlapping histological and clinical features with other malignancies, LCNEC is often misdiagnosed or identified too late, delaying effective treatment. This diagnostic uncertainty is further compounded by the disease’s aggressive nature, marked by rapid progression and a poor prognosis. Early and precise diagnosis is essential for initiating appropriate therapies that may improve survival outcomes. Failure to promptly recognize LCNEC not only denies patients access to potentially life-prolonging interventions but also worsens their already poor prognosis. To address these challenges, raising awareness among clinicians, refining pathological criteria, and utilizing advanced diagnostic tools such as molecular profiling are vital steps.

By prioritizing early and accurate diagnosis, the medical community can make significant progress in optimizing patient outcomes for this aggressive malignancy. However, the fine line between adhering to the best guideline-based approach and potential medical error underscores the complexity of decision-making in LCNEC management.

## Figures and Tables

**Table 1:** Laboratory blood test (biochemical, hematological, and coagulation markers).

Test Name	Result	Reference Range
Serum Chloride	97.70	101–109 mmol/L (Serum)
Serum Creatinine	0.92	0.70–1.20 mg/dL (Serum)
Serum Glucose	108.00	60–100 mg/dL (Serum)
Serum Potassium	4.31	3.5–5.1 mmol/L (Serum)
C-Reactive Protein (CRP)	<0.0366	0–5 mg/L (Serum)
Serum Sodium	139.30	136–146 mmol/L (Serum)
Total Serum Calcium	9.80	8.4–10.2 mg/dL (Serum)
Aspartate Aminotransferase (AST)	12.00	0–50 U/L (Serum)
Alkaline Phosphatase (ALP)	52.20	55–149 U/L (Serum)
Lactate Dehydrogenase (LDH)	215.00	135–225 U/L (Serum)
Serum Urea	32.00	10.8–38.4 mg/dL (Serum)
Quick Time (PT)	11.60	9.4–12.5 s (Plasma)
International Normalized Ratio (INR)	1.05	0.8–1.20 (Plasma)
Prothrombin Activity	93.00	70–140% (Plasma)
Activated Partial Thromboplastin Clotting Time ↘	24.20	25.1–36.5 s (Plasma)
Fibrinogen ↘	214.00	238–498 mg/dL (Plasma)
White Blood Cells (WBC) ↗	10.93	3.84–9.84 × 10^3^/μL (Blood)
Neutrophils ↗	9.20	1.54–7.04 × 10^3^/μL (Blood)
Lymphocytes	1.16	0.97–3.26 × 10^3^/μL (Blood)
Monocytes	0.56	0.18–0.78 × 10^3^/μL (Blood)
Eosinophils ↘	0.00	0.04–0.38 × 10^3^/μL (Blood)
Basophils	0.01	0.01–0.05 × 10^3^/μL (Blood)
Neutrophils % ↗	84.20	32.5–74.7% (Blood)
Lymphocytes % ↘	10.60	16.4–52.7% (Blood)
Monocytes %	5.10	4.4–12.3% (Blood)
Eosinophils %	0.00	0.0–4.0% (Blood)
Basophils %	0.10	0.1–1.2% (Blood)
Red Blood Cell Count (RBC) ↘	5.79	4.03–5.29 × 10^6^/μL (Blood)
Hemoglobin (HGB) ↗	15.70	11.0–14.5 g/dL (Blood)
Hematocrit (HCT) ↗	46.00	33.9–43.5% (Blood)
Mean Corpuscular Volume (MCV)	79.40	76.7–89.2 fL (Blood)
Mean Corpuscular Hemoglobin (MCH)	27.10	25.2–30.2 pg (Blood)
MCH Concentration (MCHC) ↗	34.10	31.8–34.8 g/dL (Blood)
Red Blood Cell Distribution Width—Standard Deviation (RDW-SD) ↘	34.20	36.7–43.8 fL (Blood)
Red Blood Cell Distribution Width—Coefficient Variation (RDW-CV)	12.10	12.4–14.5% (Blood)
Platelets (PLT)	251.00	175–332 × 10^3^/μL (Blood)
Mean Platelet Volume (MPV)	10.30	9.6–11.8 fL (Blood)
Platelet–Large Cell Ratio (P-LCR)	28.40	18.5–42.3% (Blood)
Platelet Distribution Width (PDW)	12.40	10.1–16.1 fL (Blood)

↘—decreased values relative to the normal range; ↗—increased values relative to the normal range.

**Table 2:** Immunohistochemistry panel.

Immunohistochemistry	Result
Pan-cytokeratin (AE1/AE3)	Positive
Cytokeratin 7 (CK7)	Positive
Cytokeratin 5/6 (CK5/6)	Negative
Tumor Protein 63 (p63)	Negative
Synaptophysin	Positive
Chromogranin A	Negative
CD117 (c-Kit)	Positive
S-100	Negative
CD30	Negative
Placental Alkaline Phosphatase (PLAP)	Negative
Beta-Human Chorionic Gonadotropin (Beta-HCG)	Negative
Thyroid Transcription Factor-1 (TTF1)	Negative
Napsin A	Negative
Thyroglobulin	Negative
Calcitonin	Negative
Inhibin	Negative
Glypican-3	Negative
Discovered on GIST-1 (DOG1)	Negative
Paired Box Gene 8 (PAX8)	Positive

## Data Availability

The original contributions presented in this study are included in the article. Further inquiries can be directed to the corresponding author.
